# Complete mitochondrial genome of the whitetip reef shark *Triaenodon obesus* from the British Indian Ocean Territory Marine Protected Area

**DOI:** 10.1080/23802359.2020.1775148

**Published:** 2020-06-05

**Authors:** Shaili Johri, Taylor K. Chapple, Robert Schallert, Elizabeth A. Dinsdale, Barbara A. Block

**Affiliations:** a Hopkins Marine Station, Stanford University, Pacific Grove, CA, USA; b Department of Biology, San Diego State University, San Diego, CA, USA; c Coastal Oregon Marine Experiment Station, Oregon State University, OR, USA

**Keywords:** Chagos, MPA, Genomics, BIOT, whitetip reef shark, conservation

## Abstract

We present the first mitochondrial genome of *Trianenodon obesus* from the Chagos Archipelago in the British Indian Ocean Territory (BIOT) Marine Protected Area. The mitogenome was 16,702 bp in length and consisted of 13 protein-coding genes (PCGs), 22 tRNA genes, 2 rRNA genes, and a non-coding control region (D-loop). GC content was at 38.9%. The control region was 1064 bp in length. This mitogenome for the BIOT MPA *T. obesus* differed from the previously published *T. obesus* genome by 15 bp and the differences include a 2 bp insertion and 13 single nucleotide polymorphisms distributed across the mitogenome in the BIOT MPA sequence. Whole mitogenome sequence of *T. obesus* from the Chagos archipelago presented here fills existing gaps in genetic information on marine species from the BIOT MPA and provides additional tools for species specific assessments as to the effectiveness of MPA management. In addition, methods presented here lay the framework for genetic studies in remote locations with limited infrastructure.

The Whitetip Reef Shark (*Triaenodon obesus*) is a small requiem shark with distinct white tips on its dorsal and caudal fins, and a dark gray-brown dorsal region that fades to light on the ventral side. The whitetip reef shark has a wide distribution in the Pacific and Indian Oceans. It typically lives along the bottom in clear, shallow waters surrounding coral reefs and has been reported at depths of 1,083 feet (330 m) (Smale 2019). Whole mitochondrial genomes improve the resolution of population genetic estimates and provide tools to study sub-populations of species. Here, we sequenced the complete mitochondrial genome of *T. obesus* collected from the Chagos Archipelago, British Indian Ocean Territory Marine Protected Area (BIOT MPA) (Latitude: 05.7731°S; Longitude: 071.2974°E).

The specimen was stored at the Hopkins Marine Station, Stanford University in 70% ethanol (Sample Accession # 020002232503). DNA was extracted from the muscle biopsy of an individual mature male and the mitochondrial genome was sequenced on the MinION sequencer using published methods (Johri et al. 2019; Dunn et al. 2020; Johri et al. 2020; Johri et al. 2020; Johri et al. 2020). 302,000 Fast5 files obtained from sequencing were converted to FASTQ files, and processed as described in (Johri et al. 2019), resulting in a contig of 165 reads. The mitogenome contig was then annotated as described in (Johri et al. 2020) and checked for accuracy by comparison with annotated Carcharhinidae mitochondrial genomes from GenBank including that of *Triaenodon obesus* (Genbank: NC_026287.1).

To assess the phylogenetic position of *T. obesus*, a maximum likelihood (ML) tree was generated in GeneiousVR version 8.1.9 (Kearse et al. 2012). Sixteen complete mitochondrial genomes, consisting of fifteen carcharhiniforms and two hexanchiformes, were obtained from GenBank. These sequences were aligned and phylogenetically assessed using methods described in (Johri et al. 2020).

The mitochondrial genome of *Trianenodon obesus* (GenBank: MN943497) was 16,702 bp in length and consisted of 13 protein-coding genes (PCGs), 22 tRNA genes, 2 rRNA genes, and a non-coding control region (D-loop). GC content was at 38.9%. All PCGs started with ATG and all PCGs ended with an incomplete stop codon, which are likely completed by post-transcriptional polyadenylation (Ojala et al. 1981).The control region was 1064 bp in length. This mitogenome for the BIOT MPA *T. obesus* differed from the published *T. obesus* genome (16700 bp, Genbank: NC_026287.1) (Chen et al. 2016) by 15 bp. Differences include a 2 bp insertion and 13 single nucleotide polymorphisms distributed across the mitogenome in the BIOT MPA sequence compared to NC_026287.1. Bayesian analysis (Figure 1) shows that *T. obesus* resides within the clade representing the family Carcharhinidae. Whole mitochondrial genome sequence of *T. obesus* from the Chagos archipelago presented here fills existing gaps in genetic information from the BIOT MPA. In addition, the data presented here alongwith our previously published work in the Chagos archipelago (Ferretti et al. 2018; Dunn et al. 2020; Tickler et al. 2019; Johri et al. 2020) will enable species specific assessments of top predators in the BIOT MPA and provide metrics to assess the effectiveness of MPA management. Last, methods described here lay the framework for future molecular studies in study sites with limited laboratory infrastructure. 

**Figure 1.**
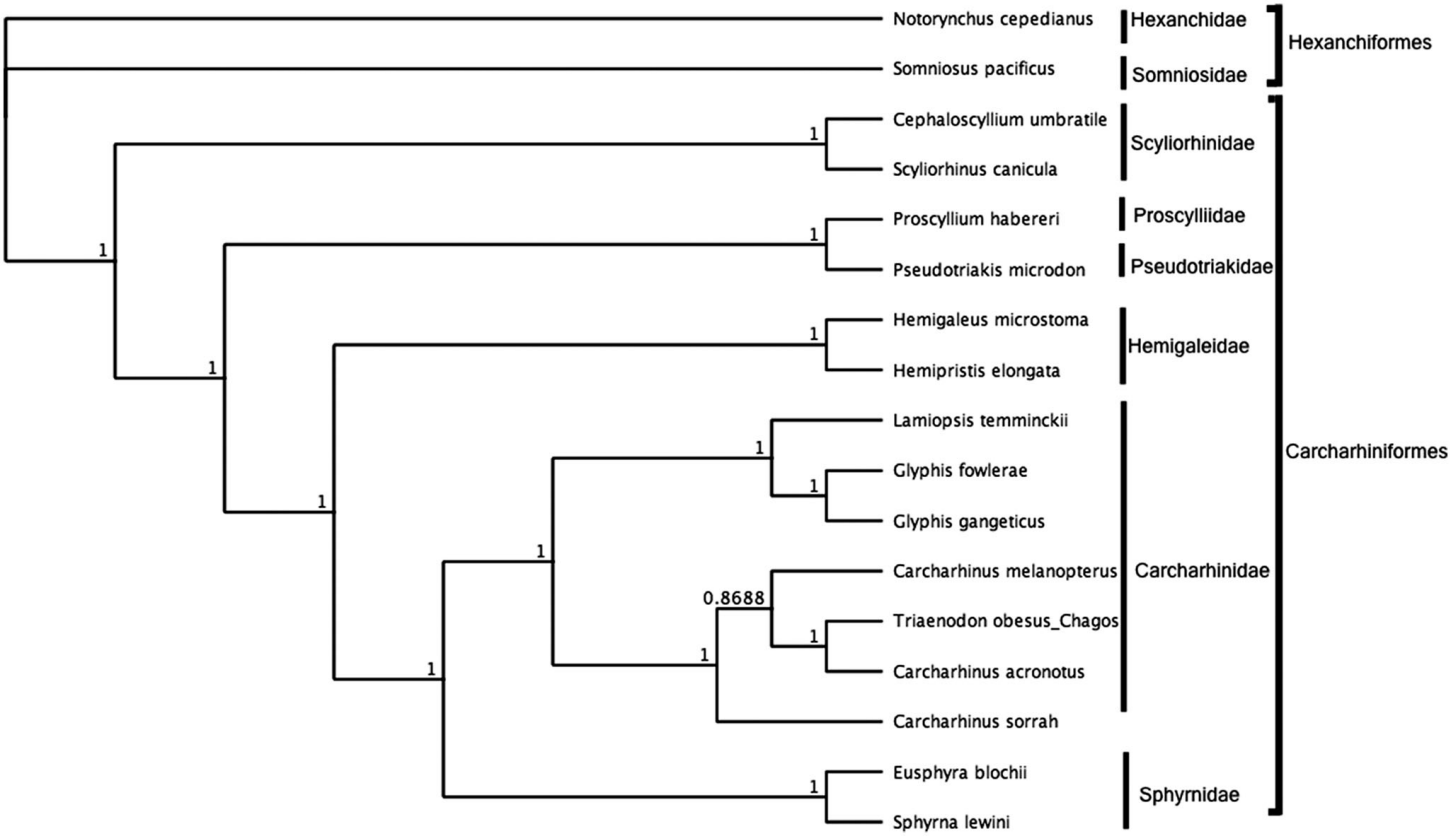
Bayesian estimate of phylogenetic position of *Triaenodon obesus* within the order Carcharhiniformes based on the complete mitochondrial genome. Members of the order Hexaniformes served as the outgroup. Families are indicated by vertical lines and orders by square brackets. Numbers at nodes are posterior probabilities. GenBank Accession Numbers: *Notorynchus cepedianus* (AB560489.1); *Somniosus pacificus* (AB560492.1); *Cephaloscyllium umbratile* (KT003686.1); *Scyliorhinus canicula* (Y16067.1); *Proscyllium habereri* (KU721838.1); *Pseudotriakis microdon* (AB560493.1); *Hemipristis elongata* (KU508621.1); *Hemigaleus microstoma* (KT003687.1); *Lamiopsis temminckii* (KT698048.1); *Glyphis fowlerae* (KT698049.1); *G. gangeticus* (KT698040.1); *Carcharhinus melanopterus* (KJ720818.1); *C. sorrah* (KF612341.1); *C. acronotus* (KF728380.1); *Triaenodon obesus*_*Chagos* (MN943497); *Eusphyra blochii* (KU892590.1); *Sphyrna lewini* (JX827259.1).

## Data Availability

Data which support the findings of this study are openly accessible in Genbank with the reference accession number MN943497.1 at DOI: https://www.ncbi.nlm.nih.gov/nuccore/MN943497.1

## References

[CIT0001] Chen X, Sonchaeng P, Yuvanatemiya V, Nuangsaeng B, Ai W. 2016. Complete mitochondrial genome of the whitetip reef shark Triaenodon obesus (Carcharhiniformes: Carcharhinidae). Mitochondrial DNA A DNA Mapp Seq Anal. 27(2):947–948. 24960567 10.3109/19401736.2014.926499

[CIT0002] Dunn N, Johri S, Curnick D, Carbone C, Dinsdale EA, Chapple TK, Block BA, Savolainen V. 2020. Complete mitochondrial genome of the gray reef shark, *Carcharhinus amblyrhynchos* (Carcharhiniformes: Carcharhinidae). Mitochondrial DNA Part B. 5(3):2080–2082. 33457750 10.1080/23802359.2020.1765208 PMC7782339

[CIT0003] Ferretti F, Curnick D, Liu K, Romanov EV, Block BA. 2018. Shark baselines and the conservation role of remote coral reef ecosystems. Sci Adv. 4(3):eaaq0333. 29532033 10.1126/sciadv.aaq0333 PMC5842041

[CIT0004] Johri S, Dunn N, Chapple TK, Curnick D, Savolainen V, Dinsdale EA, Block BA. 2020. Mitochondrial genome of the Silvertip shark, *Carcharhinus albimarginatus*, from the British Indian Ocean Territory. Mitochondrial DNA Part B. 5(3):2085–2086. 33457752 10.1080/23802359.2020.1765210 PMC7782225

[CIT0005] Johri S, Fellows SR, Solanki J, Busch A, Livingston I, Mora MF, Tiwari A, Cantu VA, Goodman A, Morris MM, et al. 2020. Mitochondrial genome to aid species delimitation and effective conservation of the Sharpnose Guitarfish (*Glaucostegus granulatus*). Meta Gene. 24:100648.

[CIT0006] Johri S, Solanki J, Cantu VA, Fellows SR, Edwards RA, Moreno I, Vyas A, Dinsdale EA. 2019. 'Genome skimming' with the MinION hand-held sequencer identifies CITES-listed shark species in India's exports market. Sci Rep. 9(1):4476. 30872700 10.1038/s41598-019-40940-9 PMC6418218

[CIT0007] Johri S, Tiwari A, Kerr EN, Dinsdale EA. 2020. Mitochondrial genome of the Smoothnose wedgefish *Rhynchobatus laevis* from the Western Indian Ocean. Mitochondrial DNA Part B. 5(3):2083–2084. 33457751 10.1080/23802359.2020.1765209 PMC7782167

[CIT0008] Kearse M, Moir R, Wilson A, Stones-Havas S, Cheung M, Sturrock S, Buxton S, Cooper A, Markowitz S, Duran C, et al. 2012. Geneious Basic: an integrated and extendable desktop software platform for the organization and analysis of sequence data. Bioinformatics. 28(12):1647–1649. 22543367 10.1093/bioinformatics/bts199 PMC3371832

[CIT0009] Ojala D, Montoya J, Attardi G. 1981. tRNA punctuation model of RNA processing in human mitochondria. Nature. 290(5806):470–474. 7219536 10.1038/290470a0

[CIT0010] Smale MJ. 2019. *Triaenodon obesus*. IUCN Red List of Threatened Species. [accessed 2019 Dec 04]. 10.2305/IUCN.UK.2005.RLTS.T39384A10188990.en.

[CIT0011] Tickler DM, Carlisle AB, Chapple TK, Curnick DJ, Dale JJ, Schallert RJ, Block BA. 2019. Potential detection of illegal fishing by passive acoustic telemetry. Anim Biotelemetry. 7(1):1.

